# The Role of Membrane-Associated E3 Ubiquitin Ligases in Cancer

**DOI:** 10.3389/fphar.2022.928794

**Published:** 2022-07-01

**Authors:** Xuankun Chen, Li Jiang, Zhesheng Zhou, Bo Yang, Qiaojun He, Chengliang Zhu, Ji Cao

**Affiliations:** ^1^ Zhejiang Province Key Laboratory of Anti-Cancer Drug Research, College of Pharmaceutical Sciences, Institute of Pharmacology and Toxicology, Zhejiang University, Hangzhou, China; ^2^ The Innovation Institute for Artificial Intelligence in Medicine, Zhejiang University, Hangzhou, China; ^3^ Center for Drug Safety Evaluation and Research of Zhejiang University, Zhejiang University, Hangzhou, China; ^4^ Cancer Center of Zhejiang University, Hangzhou, China

**Keywords:** cell membrane system, cancer, E3 ligases, drug targets, PROTAC (proteolysis-targeting chimeric molecule)

## Abstract

The cell membrane system comprises the plasma membrane, endoplasmic reticulum, Golgi apparatus, lysosome, mitochondria, and nuclear membrane, which are essential for maintaining normal physiological functions of cells. The proteins associated with these membrane-organelles are frequently modified to regulate their functions, the most common of which is ubiquitin modification. So far, many ubiquitin E3 ligases anchored in the membrane system have been identified as critical players facilitating intracellular biofunctions whose dysfunction is highly related to cancer. In this review, we summarized membrane-associated E3 ligases and revealed their relationship with cancer, which is of great significance for discovering novel drug targets of cancer and may open up new avenues for inducing ubiquitination-mediated degradation of cancer-associated membrane proteins *via* small chemicals such as PROTAC and molecular glue.

## Graphical Abstract

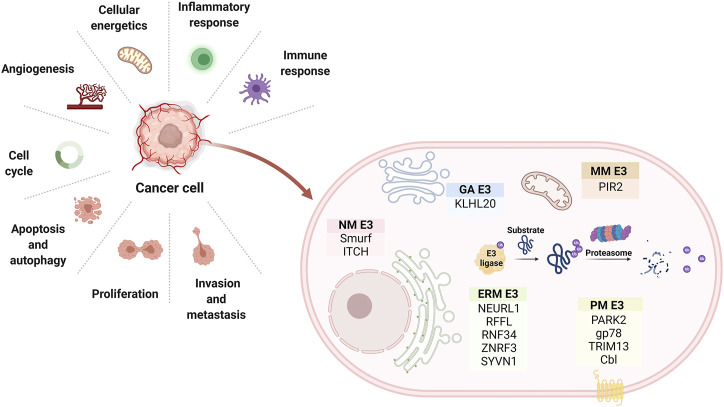

## Introduction

The cell membrane system comprises the plasma membrane, endoplasmic reticulum (ER), Golgi apparatus, mitochondria, and nuclear membrane, which is essential for maintaining cell morphology and functions. By way of illustration, plasma membrane control the substances’ entrance and exit of cells and protect the integrity of cells and maintain the shape of cells. Other membrane structures provide for the manufacture and packaging of substances within cells. Similarly, other subcellular membranes surround organelles resembling the cell membrane but with different protein and phospholipids compositions. Membrane-bound organelles provide many benefits to eukaryotic cells. Firstly, the membrane system divides the cell into multiple compartments, enabling enzymes to be concentrated in specific compartments, thereby improving the efficiency of biochemical reactions therein. Secondly, Membrane structures can protect the rest of the cell from damage by confining harmful substances to specific compartments. Thirdly, membrane-composed organelles usually rely on vesicles to transport substances and proteins (Dacks and Field, 2018). Remarkably, different membranes are well suited for their functions, primarily thanks to their various molecular composition, especially some anchor proteins. However, the dysfunction of these anchor proteins is closely associated to some human diseases like cancer (Martin and List, 2019), etc.

**FIGURE 1:**
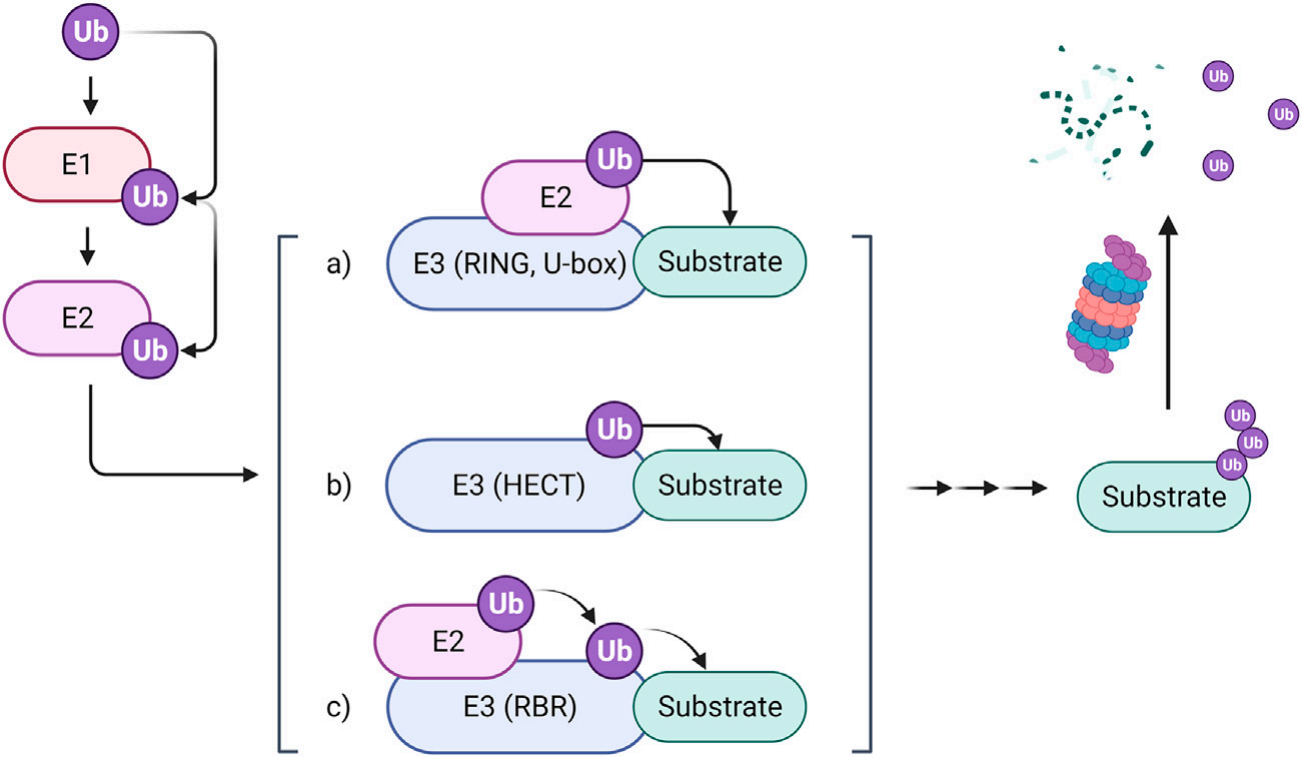
The mechanism of the ubiquitin-proteasome system. The ubiquitin-proteasome system comprises three types of enzymes. The ubiquitin-activating enzyme E1 binds with ubiquitin and then transfers it to the ubiquitin-conjugating enzyme E2, which further cooperates with three types of ubiquitin-protein ligase E3 to transport ubiquitin to substrates through different mechanisms. Finally, the labeled substrates are recognized by proteosomes for degradation.

Ubiquitylation regulates protein breakdown and signaling cascades, and hence plays a crucial role in cellular physiology. Three kinds of enzymes are required for the ubiquitylation process: E1, E2, and E3. E3 ligases, in particular, endow ubiquitination with selectivity by promoting ubiquitin transfer from an E2 enzyme to the substrate (Zheng and Shabek, 2017). So far, more than six hundred E3 ubiquitin ligases have been identified in the human proteome, which can be classified into three main types: Really Interesting New Gene (RING) E3s, Homologous to the E6-AP Carboxyl Terminus (HECT) E3s, and RING-between-RING (RBR) E3s. RING E3s are the most abundant ubiquitin ligases. They especially contain RING domains that can bind with an E2-ubiquitin thioester and promote ubiquitin cargo release. RING E3s can also stimulate the transfer of ubiquitin from E2s to the substrate directly (Deshaies and Joazeiro, 2009). The HECT E3s, containing a conserved enzymatic cysteine (Cys), accept ubiquitin from E2s and then transfer it to the substrate by a specific Lys residue (Rotin and Kumar, 2009). Another type of E3 ligases, RBR, play a role in ubiquitination through a HECT-RING hybrid mechanism. Ubiquitin is recruited to RBR *via* forming a disulfide bond with the crucial cysteine residue on RING2 and then transported to the substrate, forming an isopeptide bond with it (Figure 1). Based on the ubiquitination degradation process, E3 ubiquitin ligases have potential application value in relative researches of some proteins’ structures, functions and distribution. For instance, E3 ligases have been involved in PROteolysis-TArgeting Chimeras (PROTACs) related research to target substrate proteins for degradation. In addition, some E3 ligases were reported as novel biomarkers for some diseases like COVID-19 (Novelli et al., 2021). Furthermore, some preceding works of our group revealed that E3 ligases may participate in regulating carcinogenesis including WD repeat and SOCS box containing 1(WSB1) and Murine double minute-2 (MDM2) (Cao et al., 2015; Ying et al., 2016). These researches inspire us to shine a spotlight on the relationship between E3 ligases and cancer.

According to the statistics, E3 ligases distribute in a great many organs, cells and subcellular compartments. Compared with other subcellular localized E3 ligases, membrane-associated E3 ligases are much more crucial for the membrane-organelles, which play vital roles in maintaining their morphology and functions. For instance, mainly distributed in the mitochondrion and endoplasmic reticulum, the membrane-associated RING-CH-type finger (MARCH) proteins of E3 ubiquitin ligases positively regulate mitochondrial fission (Xu et al., 2016) and play a crucial role in controlling mitochondrial mass. It can degrade itself in dysfunctional mutants to maintain mitochondrial homeostasis and prevent cellular senescence (Bauer et al., 2017). Another E3 ligase Synoviolin (SYVN1), located in ER, stimulates the degradation of IRE1α through interaction with p53 and maintains ER function by regulating activation of the IRE1α/XBP1 pathway (Namba et al., 2015). However, the membrane-associated E3 ligases remain poorly summarized.

In this review, we summarized the 84 membrane-associated E3 ligases from the following aspects: UniProt ID, gene name, subcellular localization, E3 type and the number of transmembrane segments (Table 1). Moreover, we also described the relationship of some important E3 ligases with carcinogenesis in detail, which is significant for uncovering novel targets of cancer and may provide a new perspective of understanding the ubiquitination process, especially providing inspiration for inducing membrane protein degradation *via* ubiquitination and proteasome pathway.

**TABLE 1:** 84 membrane-associated E3 ligases.

Uniprot ID	Name	Subcellular location	Type	Transmembrane regions
Q9NZS9	BFAR	ERM	RING	4
Q9UKV5	AMFR	ERM	RING	7
Q5T197	DCST1	PM	RING	6
Q9Y4D8	HECTD4	Membrane	HECTc	1
Q96J02	ITCH	PM, cytoplasm, nucleus, EM	HECTc	NA
Q8TDB6	DTX3L	Cytoplasm, nucleus, EM, LM	RING	NA
A6NNE9	MARCHF11	VM	RING	2
Q86UD3	MARCHF3	VM, EM	RING	2
Q9P2E8	MARCHF4	GAM	RING	2
Q9NX47	MARCHF5	MM, ERM	RING	4
Q8TCQ1	MARCHF1	GAM, LM, VM, EM, PM	RING	2
Q86YJ5	MARCHF9	GAM, LM	RING	2
Q9P0N8	MARCHF2	ERM, LM, EM	RING	2
Q5T0T0	MARCHF8	VM, LM, EM	RING	2
Q86YT6	MIB1	Cytoplasm, SK, CK, PM	RING	NA
Q7L5Y9	MAEA	Cytoplasm, nucleus, PM, SK	RING	NA
O60337	MARCHF6	ERM	RING	14
Q00987	MDM2	Cytoplasm, nucleus	RING	NA
Q969V5	MUL1	MM	RING	2
Q8WY64	MYLIP	Cytoplasm, PM	RING	NA
P46934	NEDD4	Cytoplasm, PM	HECTc	NA
O76050	NEURL1	Cytoplasm, PM	RING	NA
Q6ZNB6	NFXL1	Membrane	RING	1
O60683	PEX10	Peroxisome membrane	RING	NA
O00623	PEX12	Peroxisome membrane	RING	2
O43164	PJA2	Cytoplasm, PM, ERM, GAM	RING	NA
Q8WZ73	RFFL	EM	RING	NA
O00237	RNF103	ERM	RING	4
Q9H920	RNF121	Membrane	RING	6
O60260	PRKN	Cytoplasm, nucleus, ERM, MM, PM	RING	NA
Q9H9V4	RNF122	GAM, ERM	RING	1
Q9ULX5	RNF112	Cytoplasm, nucleus, VM, PM	RING	2
Q96EQ8	RNF125	GAM	RING	NA
P29590	PML	Cytoplasm, nucleus, ERM	RING	NA
Q8WVZ7	RNF133	ERM	RING	1
Q8WU17	RNF139	ERM	RING	12
Q86XS8	RNF130	Membrane, cytoplasm	RING	1
P50876	RNF144A	PM, VM	RING	1
Q7Z419	RNF144B	MM, cytoplasm	RING	1
Q8WVD5	RNF141	Membrane	RING	NA
Q8N8N0	RNF152	LM	RING	1
Q8TEB7	RNF128	M, cytoplasm, SK, perinuclear region	RING	1
Q9H6Y7	RNF167	M	RING	1
Q8NC42	RNF149	Membrane	RING	1
Q96MT1	RNF145	ERM	RING	14
Q8N7C7	RNF148	Membrane	RING	1
Q9ULK6	RNF150	Membrane	RING	1
Q96K19	RNF170	ERM	RING	3
Q8N4F7	RNF175	Membrane	RING	5
Q96D59	RNF183	ERM, GAM, LM	RING	1
Q9NXI6	RNF186	ERM	RING	2
Q8N6D2	RNF182	Membrane, cytoplasm	RING	2
Q9Y6U7	RNF215	Membrane	RING	2
Q9NV58	RNF19A	Membrane, cytoplasm, SK	RING	2
Q6ZMZ0	RNF19B	Cytoplasmic granule membrane, ERM	RING	2
A6NCQ9	RNF222	Membrane	RING	1
E7ERA6	RNF223	Membrane	RING	1
Q96GF1	RNF185	MM, ERM	RING	2
M0QZC1	RNF225	Membrane	RING	1
Q9BY78	RNF26	ERM	RING	5
Q969K3	RNF34	PM, nucleus, cytoplasm, cytosol	RING	NA
Q9Y225	RNF24	GAM	RING	1
Q8TC41	RNF217	Membrane, cytoplasm	RING	1
Q5M7Z0	RNFT1	ERM	RING	6
Q96EX2	RNFT2	Membrane	RING	4
Q9HCE7	SMURF1	Cytoplasm, PM	HECTc	NA
A0AVI4	TMEM129	ERM	RING	3
Q9HAU4	SMURF2	Nucleus, cytoplasm, PM, membrane raft	HECTc	NA
O60858	TRIM13	ERM	RING	1
P36406	TRIM23	Cytoplasm, GAM, LM	RING	NA
Q86TM6	SYVN1	ERM	RING	6
Q8IWR1	TRIM59	ERM	RING	1
Q6ZMU5	TRIM72	PM, sarcolemma, VM	RING	NA
Q6ZT12	UBR3	Membrane	UBR	3
Q5T4S7	UBR4	Membrane	UBR	2
Q9H270	VPS11	EM, LM, VM, autophagosome	RING	NA
P49754	VPS41	EM, LM, GAM, VM, clathrin-coated vesicle	RING	NA
Q6PJI9	WDR59	LM	RING	NA
O95159	ZFPL1	GAM	RING	1
Q9H0M0	WWP1	Cytoplasm, PM, nucleus	HECTc	NA
Q8ND25	ZNRF1	VM	RING	NA
Q9ULT6	ZNRF3	PM	RING	1
Q8NHG8	ZNRF2	EM, LM, PM	RING	NA
Q8WWF5	ZNRF4	ERM	RING	1
Q9P253	VPS18	EM, LM, VM, autophagosome	RING	NA

ERM, endoplasmic reticulum membrane; GAM, golgi apparatus membrane; PM, plasma membrane; VM, vesicle membrane; EM, endosome membrane; LM, lysosome membrane; MM, mitochondrion membrane; SK, cytoskeleton and microtubule.

## ER-Localized E3

**FIGURE 2:**
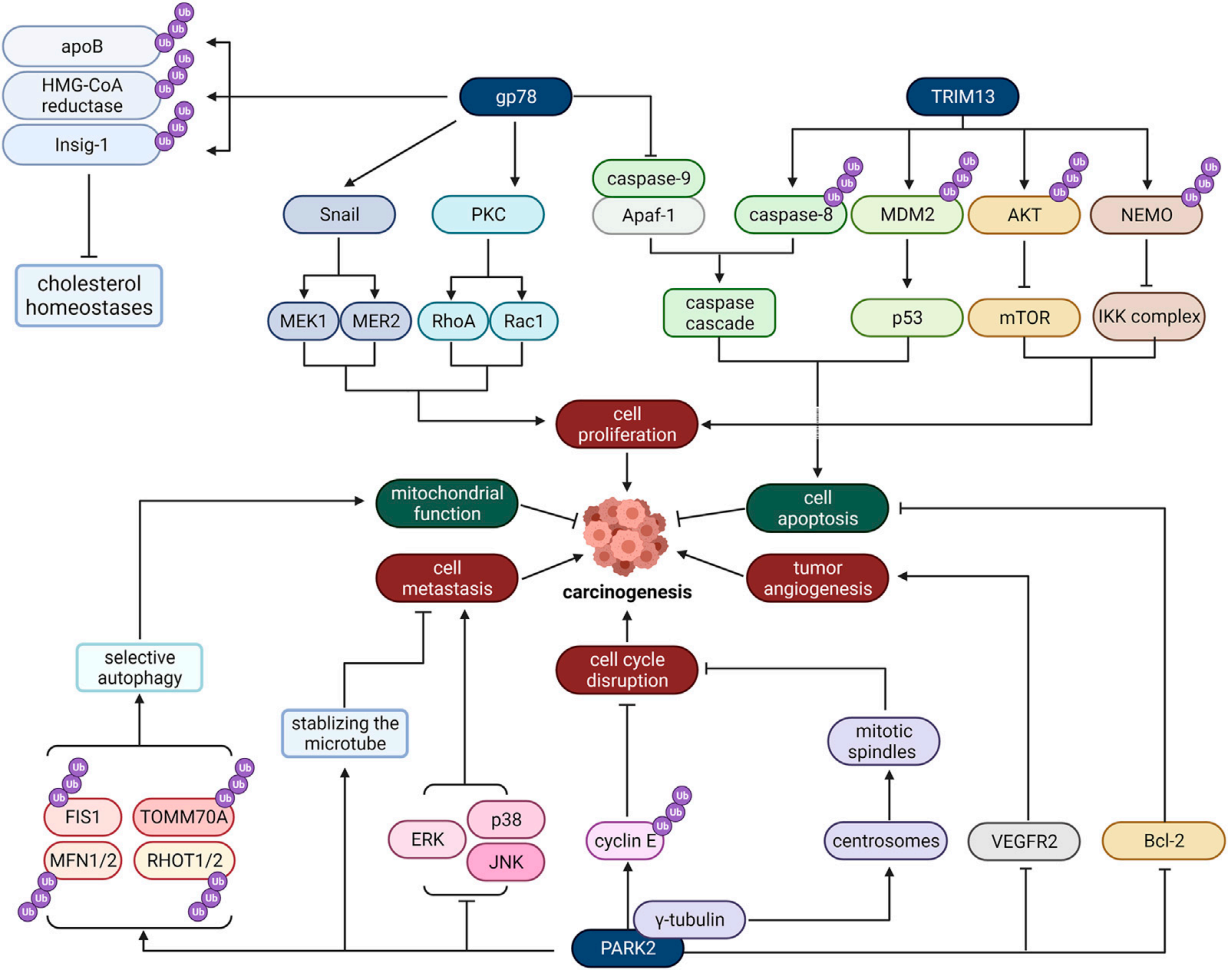
The role of some typical ER-localized E3 ligases in cancer. Gp78, TRIM13, and PARK2 are ER-localized E3 ligases. They regulate carcinogenesis through many distinct pathways, involving cell proliferation, cell apoptosis, tumor angiogenesis, cell cycle disruption, cell metastasis and mitochondrial function.

The endoplasmic reticulum functions as the transportation system of the eukaryotic cell and manufactures lipids and proteins. What’s more, the studies presented thus far provide evidence that endoplasmic reticulum stress is closely related to cancer (Yadav et al., 2014). Due to the critical role that E3 ligases play in metabolism occurred in the endoplasmic reticulum, ER-localized E3 ligases are essential for cancer development (Figure 2).

### TRIbM13

TRIM13 is a tumor suppressor gene whose deletion is common in various malignant tumors. It is a tumor suppressor in non-small-cell lung carcinoma (NSCLC) and its mRNA and protein expression was reduced in NSCLC tissues and cell lines (Xu et al., 2019). It has been reported that it is mainly located in the endoplasmic reticulum membrane, mediates the degradation of endoplasmic reticulum-related proteins, and regulates autophagy caused by endoplasmic stress (Tomar et al., 2012). So far, caspase-8, MDM2, Akt, and Nur77 have all been identified as TRIM13 substrates. Furthermore, several studies have suggested that these substrates are all related to tumor cell apoptosis and proliferation.

As for caspase-8, its ubiquitination is critical to proceed downstream caspase cascade, which results in cell apoptosis. TRIM13-induced caspase-8 ubiquitination may lead to its transportation to autophagosomes. Autophagosomes anchor caspase-8 and fusion with lysosomes, providing an environment for cleavage and activation of caspase-8. When the endoplasmic reticulum is stressed, the cell will amplify the downstream caspase cascade and cause cell death (Tomar et al., 2013).

Besides caspase-8, TRIM13 can also form complexes with MDM2, which negatively regulates the tumor suppressor p53 and AKT to provoke cell apoptosis. As a result of their interaction, MDM2 and AKT are ubiquitinated and degraded by proteasomes, thereby increasing the stability of p53, reducing the AKT kinase activity, and inducing cell apoptosis consequently (Joo et al., 2011).

This study suggests that TRIM13 can promote tumor cell apoptosis, which indicates that TRIM13 may be deleted or inactivated in some cancer tissues and cell lines. In 2019, the hypothesis was confirmed by Xu et al. (2019) experimentally. They found that compared with non-cancerous tissues and normal bronchial epithelial cell lines, non-small cell lung cancer tissues and cell lines have reduced TRIM13 mRNA and protein expression. And it is also been found that TRIM13 partly induces the apoptosis of NSCLC cells under the mediation of caspase-3 and exerts an antitumor effect.

TRIM13 can not only promote tumor cell apoptosis but also regulate cell proliferation. In 2014, Tomar and Singh (2014) investigated the influence of TRIM13 on cell growth and proliferation. According to their research, NEMO, a substrate of TRIM13, plays a notable role in the NF-κB signaling pathway, which regulates the expression of several inflammatory cytokines and is associated with cancer. TRIM13 initially interacts with NEMO to induce its ubiquitination and degradation, which may suppress the activity of the IKK complex and thus hinder NF-κB signaling. Ultimately, TRIM13 inhibits the proliferation of cancer cells.

### Gp78

Gp78, commonly known as autocrine motility factor receptor (AMFR), was initially found in the B16-F1 melanoma cell line (Nabi and Raz, 1987) and has been discovered to be an AMFR essential for tumor metastasis and migration (Watanabe et al., 1991). The ligand for gp78 is AMF (Nabi et al., 1990), an extracellular tumor cytokine. In response to AMF stimulation, gp78 is activated and then changes cell adhesion, proliferation, movement, and apoptosis activities by activating the downstream signaling pathway. Specifically, the activation of gp78 triggers a protein kinase C-dependent signal cascade and then activates and upregulates RhoA and Rac1 (Kanbe et al., 1994; Tsutsumi et al., 2002), which induce the reorganization of the lesion contact and the formation of stress fibers, resulting in enhanced cell motility and proliferation. The activated gp78 also upregulates the transcription factor SNAIL, leading to loss of cell adhesion (Silletti et al., 1996; Tsutsumi et al., 2004), and phosphorylates MEK1 and MEK2 to activate the MAPK pathway and increases cell proliferation (Torimura et al., 2001). Besides, the expression level of Apaf-1 and caspase-9 is reduced when gp78 is activated, which may inhibit tumor cell apoptosis (Haga et al., 2003).

Other than the function mentioned above of gp78 as a receptor, it can ubiquitinate and degrade some proteins as an E3 ligase, which is associated with its locations. Gp78 is internalized through the endocytosis pathway and directly enters the smooth ER when combined with AMF. Therefore, gp78 is located on the plasma membrane, smooth ER closely connected with mitochondria, and a small amount of rough ER. In ER, gp78 is crucial in proteasomal degradation of ERAD-targeted proteins, and some substrates identified so far contain cholesterol homeostases, such as apolipoprotein B (Liang et al., 2003), HMG coenzyme A reductase (Song et al., 2005) and Insig-1 (Lee et al., 2006). Other gp78 substrates like KAI1, a metastasis inhibitor, are closely correlated with sarcoma metastasis (Tsai et al., 2007).

### PARK2

PARK2 is a tumor suppressor gene that plays an essential part in cancer progression. It is related to microtubule stability, cell cycle disruption, mitochondrial homeostasis, cell apoptosis, and metabolism that regulates cell state (Manzanillo et al., 2013). Current studies have confirmed loss of function of PARK2 in various human cancers, including breast (Rodriguez et al., 2000), ovarian (Saito et al., 1996)_,_ lung (Kong et al., 2000), and renal cancers (Morita et al., 1991), implying that its inactivation may promote tumor transformation and progression. What’s more, PARK2 protein is negatively regulated in many primary tumors, which may contribute to cancer development. For instance, Parkin deficiency may promote pancreatic tumors through dysregulation of microtubule-dependent mitotic kinesin Eg5 expression and subsequent spindle behavior defects (Sun et al., 2013).

Microtubule stability is critical for cell proliferation, and PARK2 may inhibit tumor migration by stabilizing microtubules through an E3-independent way when paclitaxel is administered as an anticancer drug. This effect is triggered by the binding of three microtubule-binding domains of Parkin (MAPKs), which are mitogen-activated protein kinases, to microtubules. By binding to the external layer of microtubules, Parkin enhances microtubule-paclitaxel interaction as well as the activity of paclitaxel, thereby promoting microtubule stability and assembly, which are two hallmarks of paclitaxel cytotoxicity. Therefore, PARK2 levels may predict paclitaxel treatment outcomes in breast cancer (Wang et al., 2009). In addition, the following activation of protein kinases associated with microtubules such as p38, ERK, and JNK is also blocked, thereby antagonizing the effects of microtubule depolymerization drugs like colchicine (Ren et al., 2009).

Carcinogenesis may be incited by cell cycle disruption induced by Parkin dysfunction. A seminal study of PARK2 by Kyoko Ikeuchi (2009) reports that Parkin help with the ubiquitination and proteasome degradation of cyclin E in human colon cells (Ikeuchi et al., 2009). Furthermore, Shiam-Peng (2010) demonstrated that PARK2 contributes to cell cycle arrest and growth inhibition by specifically upregulating CDK6 mRNA levels in MCF7 breast cancer cells (Tay et al., 2010). Multiple shreds of evidence suggest that PARK2 also modulates centrosomes and mitotic spindles *via* interacting with γ-Tubulin (Chen et al., 2012). Since centrosomes contribute to mitotic spindles formation, their inactivation may contribute to cell division dysregulation.

Mitochondrial function is usually impaired in cancer, and PARK2 is implicated in mitochondrial functional regulation and turnover. PARK2 combines with mtDNA to enhance mitochondrial transcription mediated by TFAM and restore the expression of PGC-1α, thus promoting mitochondrial production (Kuroda et al., 2006). In addition, it protects mitochondrial genomes from reactive oxygen species-induced damage, and it is also involved in mtDNA repair (Rothfuss et al., 2009). What’s more, activated PARK2 acts as a catalyst for rapid ubiquitination of various mitochondrial proteins, such as FIS1, MFN1/2, TOMM70A, RHOT1/2, etc. (Chan et al., 2011; Narendra et al., 2012; Sarraf et al., 2013), which then recruits adaptor proteins to initiate selective autophagy. Ultimately, PARK2-reliant mitochondrial autophagy maintains healthy mitochondrial populations by selectively degrading damaged mitochondria. Therefore, PARK2 is vital for promoting mitochondrial function and maintaining mitochondrial genome integrity and popularity. As a result, changes in PARK2 can be associated with tumorigenesis.

PARK2 also regulates the activity of several apoptosis-related proteins of the Bcl-2 family, including Bax, Bcl-2, and Mcl1 (Chen et al., 2010; Johnson et al., 2012; Ekholm-Reed et al., 2013), promoting apoptosis of tumor cells. In addition, PARK2 boosts cell apoptosis produced by Microtubule stabilizers and HDAC inhibitors in liver and breast tumor cells, according to studies (Wang et al., 2004; Wang et al., 2009). Furthermore, PARK2 makes HeLa cells susceptible to apoptosis induced by TNF-α (Lee et al., 2012).

PARK2 may influence cancer cell metabolism to some extent. For example, PARK2 is the target gene of p53, which regulates energy metabolism. PARK2 deficiency enhances glycolysis while decreasing mitochondrial respiration, leading to the Warburg effect (Zhang et al., 2011). Moreover, in gliomas, the EGFR-Akt pathway is negatively regulated by PARK2, and PARK2 overexpression can inhibit signal transduction through Akt/mTOR. And the loss of parkin function will enhance the expression of cyclin D1 and Akt-related growth promotion signals and, at the same time, promote the proliferation of glioma cells. Besides, in gliomas PARK2 downregulates VEGFR2, a high-affinity tyrosine kinase receptor involved in tumor angiogenesis. Therefore, it may have the effect of inhibiting tumor angiogenesis (Yeo et al., 2012).

### SYVN1

SYVN1 inhibits breast cancer growth and metastasis through the miR-96-5p/SYVN1 axis (Gao et al., 2018). It interacts with IGF-1R and promotes its ubiquitination and degradation through the proteasome. This, in turn, leads to inhibition of the growth, migration and invasion of breast cancer cells (Xu et al., 2015). Tumor metastasis is closely associated with the poor prognosis of hepatocellular carcinoma (HCC). Some studies have found that E3 ubiquitin has been analyzed by proteomics and ubiquitinomics of HCC. Furthermore, there is a correlation between SYVN1 and tumor metastasis. Moreover, SYVN1 interacts with heat shock protein 90 and contributes to the ubiquitination of eukaryotic elongation factor 2 kinase. (Ji et al., 2021).

Tumor cell metabolic abnormalities may cause ER lesion and unfolded protein response (UPR), which maintains ER homeostasis by inducing degradation of unfolded proteins. Typically, proteins not properly folded are perceived by adaptor proteins such as chaperones, ubiquitinated by E3 ubiquitin ligases and degraded by proteasomes. However, ER stress would induce cell apoptosis to exclude them from normal cells. Therefore, ER function is critical for the survival of cancer cells.

## Plasma Membrane-Localized E3

**FIGURE 3:**
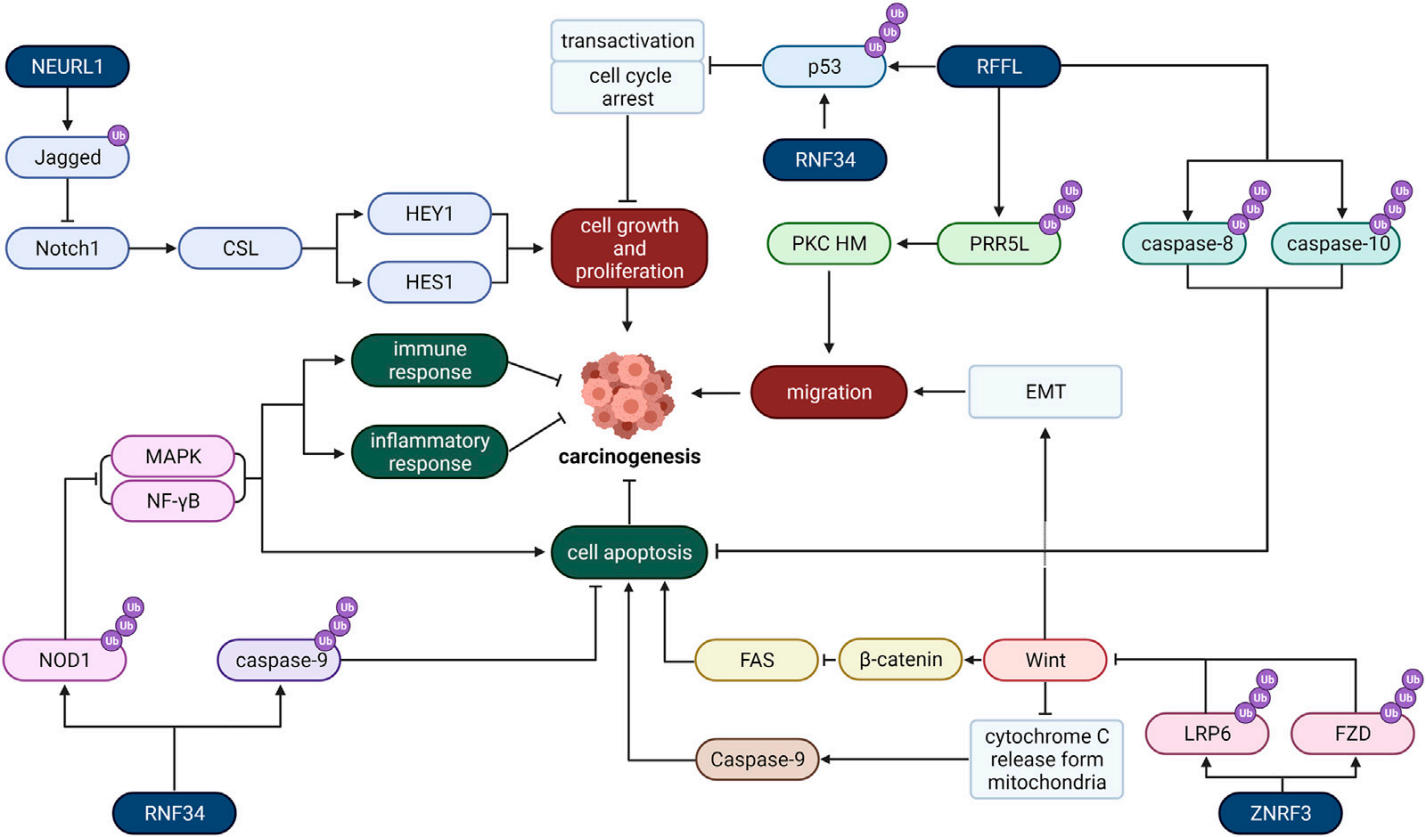
The role of typical Plasma membrane-localized E3 ligases in cancer. RNF34, REFL, RNF34, and NEURL1 are four plasma membrane-localized E3 ligases. They regulate carcinogenesis through various pathways, including cell growth and proliferation, migration, cell metastasis, apoptosis, inflammatory response, and immune response.

Plasma membrane not only regulates the flow of substances into and out of cells but also protects the integrity of cells and keeps their form. Multitudes of researches have proved that frequent mutations of plasma membrane-associated E3 ligases are engaged in carcinogenesis (Figure 3).

### ZNRF3

Zinc and ring finger 3 (ZNRF3) is located in the plasma membrane, which is usually correlated to cancer development. In 2014, Assié et al. (2014) reported frequent mutations of ZNRF3 in adrenocortical carcinoma (ACC). Compared to normal surrounding tissues, the ZnRF3 level in gastric tumor tissues has been lower (Zhou et al., 2013), and ZnRF3 mutations are prevalent in pancreatic cancer (Wolpin et al., 2014). Numerous literature has investigated how ZNRF3 regulates the Wnt/β-Catenin signal, which is correlated with cancer (Clevers and Nusse, 2012) because Wnt helps the stabilization and nuclear localization of β-Catenin, which contributes to the formation of TCF/β-Catenin complex and recreation of other co-activators to promote gene activation like c-MYC and cyclin D1 (Ma et al., 2012).

Ubiquitination-mediated Wnt receptor turnover has become a key regulating factor of the Wnt pathway because it affects the sensibility of cells to Wnt ligands. And ZNRF3 precisely regulates Wnt pathway activity by promoting the degradation of Wnt receptors. ZNRF3 inhibits Wnt/PCP and Wnt/β-Catenin signaling pathway by promoting the ubiquitination and degradation of Wnt protein core receptor FZD (Jiang et al., 2015) and Wnt protein co-receptor LRP6 (Hao et al., 2012; Koo et al., 2012). Therefore, ZNRF3 negatively regulates the Wnt pathway and may inhibit cancer cell growth.

It has also been found that ZNRF3 hinders the growth of cancer cells and promotes cell apoptosis by regulating the Wnt/β-Catenin/TCF signaling pathway (Zhou et al., 2013) for the reason that Wnt-1 inhibits apoptosis by blocking mitochondrial cytochrome C release, thereby inhibiting the activity of caspase-9 (Li et al., 2006; Brocardo and Henderson, 2008). And other studies have shown that Wnt signaling inhibits cancer cell apoptosis by impelling NF-κB. Activated β-Catenin may decrease the expression level of FAS, targeted by NF-κB, whence possibly suppressing Fas-mediated apoptosis, leading to tumorigenesis (Ma and Hottiger, 2016).

Furthermore, ZNRF3 may also restrain the migration and invasiveness of some cancer cells like papillary thyroid carcinoma cells by this pathway (Deng et al., 2015). Previous research has established that epithelial-mesenchymal transition (EMT) is a critical step in the migration of cancer cells from the original cancer lesions to nearby organs (Christofori, 2006). The Wnt/β-Catenin pathway is one mechanism for cells to undergo EMT (Thiery et al., 2009). Consequently, ZNRF3 peradventure inhibits cancer cell invasion.

### RFFL (CARP-2)

The ring finger and FYVE-like (RFFL) domain-containing E3 ligase is a member of caspase 8/10-associated RING proteins (CARPs), so it is also named CARP-2. Studies have found that RFFL inhibits p53, which is an effective tumor suppressor that tends to be mutated in tumor cells (Bode and Dong, 2004; Olivier et al., 2004) and promotes the growth of cancer cells. RFFL has been reported to have strong functions in colorectal cancer cells (Dong et al., 2013). In contrast, CARP’s silencing upregulates p53 expression and facilitates transcriptional activation and tumor suppression. CARPs negatively regulate p53 through ubiquitination and proteasome degradation of p53 and inhibit transactivation and cell cycle arrest. CARPs can target not only non-phosphorylated p53 but also phospho-p53^ser20^ and possibly phospho-p53^ser15 74^, both of which are accumulated after DNA damage and cannot be ubiquitinated by other p53 associated E3s like MDM2. Consequently, CARPs are critical to regulating cancer cell growth through the p53 pathway.

Apart from promoting cancer cell proliferation, CARPs also inhibit cancer cell apoptosis. It helps the ubiquitin-mediated proteolysis of death effector domain (DED) caspase, which induces the damage of cell target during apoptosis and is challenging to be inhibited by regular inhibitors of apoptosis proteins (IAP). It has been found that CARPs interact specifically with caspase-8 and caspase-10 (McDonald and El-Deiry, 2004) and induce their ubiquitination and degradation *via* the proteasome pathway, thereby impeding cancer cell apoptosis.

And RFFL plays a pivotal part in cell migration. RFFL induces ubiquitination and degradation of PRR5L, which leads to mTORC2-mediated phosphorylation and activation of PKC HM. Continuous activation of PKC is indispensable for maintaining tumor cell migration. Therefore, RFFL may be a potential target to promote tumor migration and metastasis (Gan et al., 2013). Hence, the regulation of RFFL is essential for tumors. Studies have reported that miR-133a can straight bind to RFFL mRNA and inhibit its translation, thereby reducing the level of RFFL protein, enhancing cell apoptosis and inhibiting cell proliferation (Dong et al., 2013).

### RNF34 (CARP-1)

Belonging to the CARP family, RNF34 induces the ubiquitination and degradation of death effector domain (DED) caspase to suppress cancer cell apoptosis (McDonald and El-Deiry, 2004). And RNF34 can also inhibit cancer cell apoptosis by regulating the NOD1 pathway. NOD1 can not only induce cell apoptosis but also control cell proliferation, and NOD1 activation can lead to caspase-8 and caspase-9-induced apoptosis (da Silva Correia et al., 2007). Recently there have been reports of missing NOD1 and breast tumor growth (da Silva Correia et al., 2006). RNF34 mainly affects the NOD1 pathway negatively. It interacts directly with NOD1 to induce its ubiquitination and subsequent degradation, therefore reducing the expression level of NOD1 in the cell. As a result, RNF34 hinders NOD1-mediated apoptosis. And it inhibits the activation of NOD1-dependent nuclear factor-κB (NF-γB) along with mitogen-activated protein kinase (MAPK), which results in cytokine, chemokine, antimicrobial peptide production and apoptosis when activated (da Silva Correia et al., 2007; Hasegawa et al., 2008; Park et al., 2007). Therefore, it may inhibit the cellular immune response, inflammatory response and apoptosis in this way.

In addition, RNF34 also stimulates the degradation of p53 and then hinders transactivation, and cell cycle arrests like other CARP members, such as RFFL mentioned above (Yang et al., 2007), which means it also regulates cancer cell cycles and may accelerate cell growth and proliferation.

### NEURL1

Neuralized-like protein 1 (NEURL1) is a conserved E3 ligase investigated as a candidate tumor suppressor. The subcellular localization of NEURL1 has not been definitively determined, while some studies demonstrated that overexpressed NEURL1 is localized in the plasma membrane in an N-myristylation-dependent manner (Koutelou et al., 2008). According to studies, NEURL1 is significantly down-regulated in medulloblastoma cells through histone modification and exhibits various tumor suppressor properties, including promotion of cell apoptosis and suppression of tumor growth, angiogenesis, and invasion (Koutelou et al., 2008). These effects are mainly produced through the regulation of the Notch signaling cascade.

NEURL1 can function as an E3 ubiquitin ligase to monoubiquitinate the extracellular domain of a membrane-tethered protein Jagged1 and trigger the endocytosis and degradation of Jagged1 (Koutelou et al., 2008). Decreased Jagged1 level weakens signaling between Jagged1 and its receptor, Notch1, on the surfaces of neighboring cells. This results in less cleavage and detaching of Notch intracellular region (icN1) from the membrane and binding with CSL transcription factor in the nucleus (Sjölund et al., 2005). Consequently, inhibition of the Notch1 signaling pathway downregulates the transcriptional initiation of a variety of cell cycle-related proteins, including HES1 and HEY1 (Teider et al., 2010), suppressing the growth and division of tumor cells.

Other upstream proteins may regulate this effect of NEURL1. Fe65 facilitates the recruitment of NEURL1 to form a stable ternary complex with Jagged1 and is considered a factor negatively regulating the Jagged1/Notch1 pathway (Lee et al., 2015). Neuritin interacted with NEURL1 and down-regulates NEURL1 expression. Therefore, it is considered a positive regulator of the Jagged1/Notch1 pathway (Zhang et al., 2017).

### Cbl

Cbl proteins mainly exist on the cell membrane surface and play a significant role in tumorigenesis and antitumor immunity (Liyasova et al., 2015). And it is been reported that Cbl expression correlates with human colorectal cancer (Kumaradevan et al., 2018) and it may inhibit lung cancer and glioblastoma cells’ migration (Lee et al., 2018). First, its activity as an E3 ligase negatively regulates signalings by activated RTKs: this family of proteins attenuates receptor tyrosine kinases by ubiquitinating RTKs, targeting them to lysosomes for degradation (RTK)-related signaling (Ma et al., 2013). Nevertheless, Cbl’s E3 function is lost due to cancer mutations. Furthermore, Cbl-mediated ubiquitination can alter the cellular localization of the protein to modulate its function. In addition, Cbl also act as adaptors to active RTKs by recruiting signaling molecules. For example, it has been shown that Cbl act as adaptors to recruit PI3K to activate RTKs, which subsequently activate the PI3K/AKT pathway (Dombrosky-Ferlan and Corey, 1997; Ueno et al., 1998).

Cbl-b negatively regulates the antitumor function of T cells and natural killer (NK) cells through its E3 ligase activity. Therefore, deletion of Cbl-b will potentiate the NK cell activity. For example, Cbl-b deficient NK cells can suppress oncogene-driven breast cancer. The tyrosine kinase receptor TAM family is the molecular substrate of Cbl-b, including Tyro3, Axl, and Mer. And TAM receptors also negatively regulate NK cells. Therefore, the Cbl-b/TAM receptors inhibit NK cell activation (Paolino et al., 2014), and the consequences of Cbl protein deficiency can lead to malignant tumors or immune dysfunction.

## Golgi Localized E3 Ligases

**FIGURE 4:**
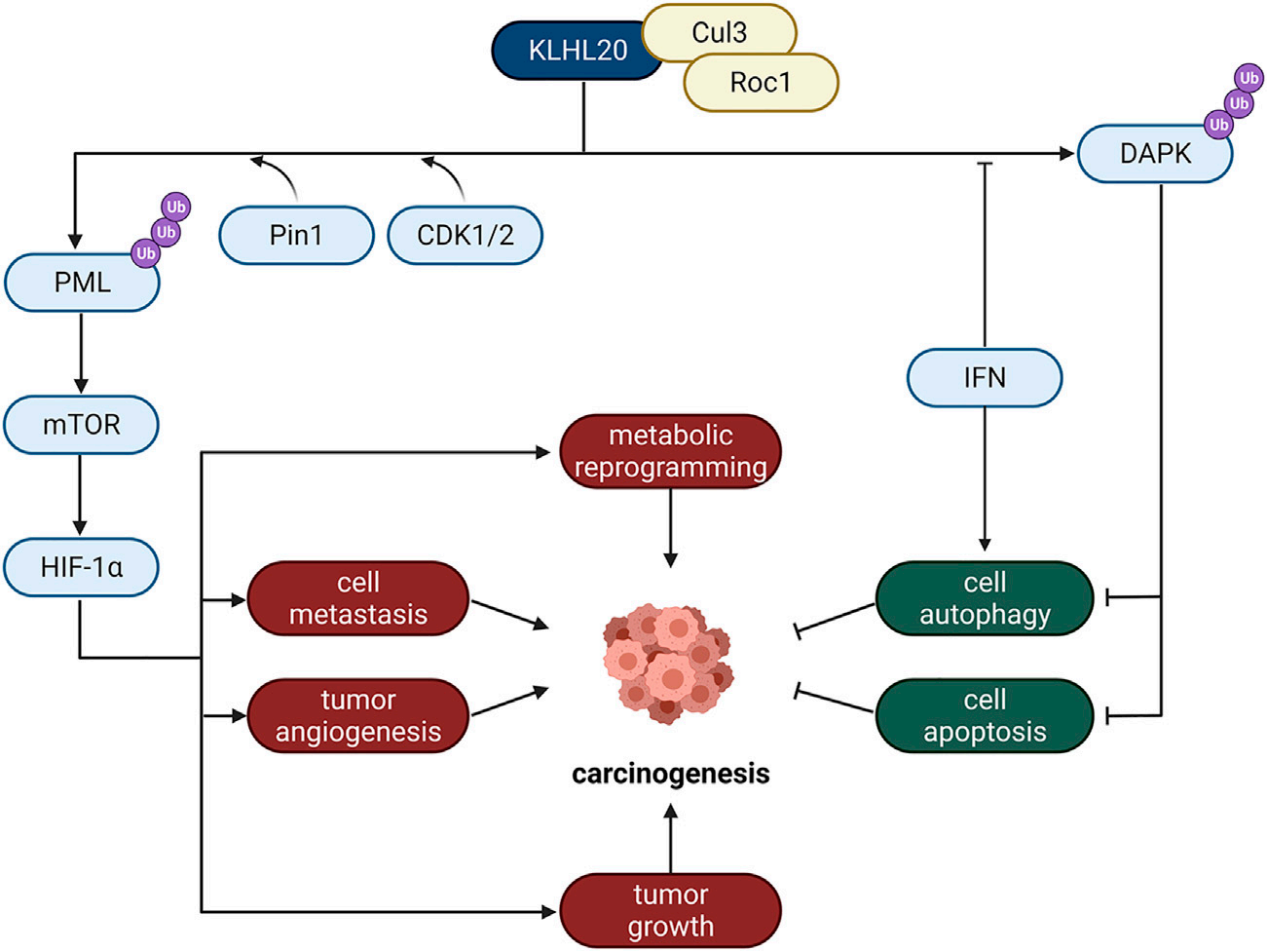
The role of Golgi localized E3 ligases in cancer. KLHL20 is an important Golgi E3 protein, which regulates carcinogenesis through many different pathways, including metabolic reprogramming, cell metastasis, tumor angiogenesis and tumor growth.

The Golgi apparatus is of particular importance for secretory proteins. Its significant function of regulating metabolism mainly dependents on membrane fluidity and abundant important anchored proteins. And Golgi localized E3 ligases are closely related to tumor hypoxia responses and cell apoptosis, such as KLHL20 (Figure 4).

KLHL20, a Cullin3 (Cul3) substrate adaptor, belongs to Broad-Complex, Tramtrack, and Bric-a-brac (BTB) family (Pintard et al., 2004). Only if it forms an E3 ligase complex with Cul3 and Roc1 can it induce the ubiquitination of its substrates like PML and DAPK. It promotes tumor growth, migration, angiogenesis and metabolic reprogramming as well as inhibits the processes of autophagy and apoptosis of cancer cells by promoting substrate ubiquitination and degradation, thus promoting prostate cancer progression (Yuan et al., 2011).

KLHL20 mediates hypoxia-induced PML ubiquitination and proteasome-induced degradation. CDK1/2 and Pin1 are also implicated in the regulation of this pathway. CDK1 and CDK2 promote KLHL20-mediated degranulation of PML, and Pin1-mediated proline isomerization promotes the recruitment of PML to KLHL20. Because PML negatively regulates hypoxia-inducible 1α (HIF-1α) protein synthesis by inhibiting mTOR activity (Bernardi et al., 2006). Therefore, degradation of PML would increase HIF-1α content, thereby promoting a variety of HIF-1α-regulated metabolic reprogramming, migration, tumor growth and angiogenesis, etc. (Brahimi-Horn et al., 2007; Finger and Giaccia, 2010; Semenza, 2010).

Studies have shown that KLHL20 also enhances DAPK ubiquitination and proteasome degradation to inhibit cell apoptosis. Death-associated protein kinase (DAPK) is a tumor suppressor. Moreover, interferon (IFN) can suppress tumor cell proliferation and trigger apoptosis, and it has been used in cancer therapy. Because DAPK is a pivotal factor of IFN-induced autophagy (Inbal et al., 2002), KLHL20 impairs its proapoptotic function. It was also found that IFN could reduce the efficiency of KLHL20-DAPK complex formation by sequestering KLHL20, thus reducing DAPK ubiquitination by KLHL20 and stabilizing DAPK protein content; as a result, aiding IFN-induced autophagy (Inbal et al., 2002).

## Mitochondria Localized E3

PIR2/RNF144B is a potential targeted biomarker in endometrial cancer to promote proliferation and is mainly localized on the mitochondrion membrane. Experiments in human osteosarcoma cells Saos-2 revealed that the expression of apoptin issues in the activation of proapoptotic TAp73, ending in the upregulation of PUMA and cell death. Thus, PIR2 degrades anti-apoptotic ΔNp73 to increase TAp73 stability to induce apoptosis. In brief, it induces ubiquitination of ΔNp73 for degradation and activates the TAp73-mediated apoptosis pathway. And it was previously shown to regulate the stability of p21WAF1 or p63, which mediate cell growth arrest and epithelial homeostasis (Conforti et al., 2013).

## Nuclear Membrane-Localized E3

### ITCH

ITCH is localized on the cell and nuclear membranes. It plays a fundamental role in different cellular contexts depending on its different substrates. Its substrates are mainly divided into two categories: transcription factors and cell growth factors, like ErbB-4, a member of the EGFR/ERbB family. These substrates’ ubiquitination and proteasomal degradation affects cell growth, differentiation and apoptosis, and is associated with tumors malignant transformation and chemoresistance (Melino et al., 2008). And it is been found that ITCH is a downstream target of miR-10b which promoted melanoma progression by repressing ITCH (Wang et al., 2019).

### Smurf1 and Smurf2

Smad ubiquitin regulatory factor 1 (Smurf1) and Smurf2 are E3 ligases that inhibit the TGF-β pathway through ubiquitination-induced receptors for degrading smads and transforming growth factor-β (TGF-β). Smurf1 may promote ovarian cancer invasion and epithelial-to-mesenchymal transition (EMT) (Fan et al., 2019) and Smurf2 may motivate colon cancer cell proliferation (Yu et al., 2019). However, their substrates are different. Smurf1 binds to Smad1 and Smad5, while Smurf2 binds to Smad1, Smad2, Smad3, Smad6, and Smad7. Smurf2 enhanced the repressive activity of Smad7 while decreasing the transcriptional activity of Smad2. Smurf2 can bind to the transcriptional co-repressor SnoN and degrade it through Smad2 (Bonni et al., 2001). Therefore, Smurf2 may enhance TGF-β signaling beneath certain circumstances. In addition, due to differences in post-translational regulation, the functions of smurf2 and smurf1 also differ to some extent. At the same time, Smurf1 can regulate cell motility by promoting the ubiquitination and degradation of RhoA (Wang et al., 2003), which plays a certain role in the development of cancer. On MDA-MB 231 cells, Smurf2 can ubiquitinate and subsequently degrade Smurf1, but not vice versa. The knockdown of Smurf2 leads to an increase in the protein level of Smurf1, which promotes cell migration *in vitro* and bone metastasis *in vivo* (Fukunaga et al., 2008).

## Conclusion and Prospects

In summary, membrane-associated E3 ligases are of great significance to the therapeutic improvement of cancer. Lipid alterations in membrane and other cancer syndromes in cancer cells are closely related to E3 ligases (Zalba and ten Hagen, 2017). Thus, the membrane system regulates the proliferation, migration and differentiation of cancer cells partly *via* E3 ligases. Increasing evidence indicates that specific E3 ligases play a key role in carcinogenesis. What’s more, some membrane-associated E3 ubiquitin ligases are observed to be frequently mutated in many human cancer cell lines, which may induce chemoresistance and cause poor clinic prognosis. These research findings would eventually shed light on a new class of antitumor drugs targeting E3 ubiquitin ligases and the research and development of sensitive biomarkers for cancer diagnosis, treatment, and prognosis, which requires further constant exploration. What’s more, several studies have demonstrated that some E3 ubiquitin ligases are related to the regulation of innate immune response, which provides a solid theoretical basis for expanding the application of E3 ligases to promote tumor immunotherapy.

In the future, the rapid development and breakthrough of PROTAC may largely depend on some research on membrane-associated E3 ligases. Some membrane-anchored proteins, for example, are difficult to target using targeted degradation techniques, but a new strategy utilizing membrane-associated E3 ligases may help the ubiquitination and degradation of these proteins. In addition, the cellular trafficking system may deliver some proteins from the plasma membrane to the trans-Golgi network, thereby chewing up these proteins.

Consequently, the membrane-associated E3 ligases have crucial functions and potential application values in developing new therapeutic strategies for cancer. It is anticipated that membrane E3 ligases will soon be of great significance in tackling medical and biological research problems with more in-depth research and understanding of membrane-associated E3 ubiquitin ligases.
